# Adsorption Behaviour of Pb and Cd on Graphene Oxide Nanoparticle from First-Principle Investigations

**DOI:** 10.3390/ma17122831

**Published:** 2024-06-10

**Authors:** Preslie Sala Nianga-Obambi, Dick Hartmann Douma, Anne Justine Etindele, Abdulrafiu Tunde Raji, Brice Rodrigue Malonda-Boungou, Bernard M’Passi-Mabiala, Stephane Kenmoe

**Affiliations:** 1 Groupe de Simulations Numériques en Magnétisme et Catalyse, Faculté des Sciences et Techniques, Université Marien Ngouabi, Brazzaville BP 69, Congo; cohenobambi@gmail.com (P.S.N.-O.); malondabrice@gmail.com (B.R.M.-B.); 2 Higher Teachers Training College, University of Yaounde I, Yaounde P.O. Box 47, Cameroon; anne.etindele@univ-yaounde1.cm; 3 Center for Augmented Intelligence and Data Science (CAIDS), College of Science, Engineering and Technology (CSET), University of South Africa (UNISA), UNISA Muckleneuk Campus, Preller Street, Muckleneuk, Pretoria 0003, South Africa; rajiat1@unisa.ac.za; 4 Institut National de Recherches en Sciences Exactes et Naturelles (IRSEN), Brazzaville BP 2400, Congo; bmpassimabiala@gmail.com; 5 Department of Theoretical Chemistry, University of Duisburg-Essen, Universität Str. 2, 45141 Essen, Germany

**Keywords:** graphene oxide, density functional theory, heavy metals, adsorption

## Abstract

Graphene oxide (GO) is considered as a promising adsorbent material for the removal of metal from aqueous environments. Here, we have used the density functional theory (DFT) approach and a combination of parameters to characterise the interactions of GO with lead (Pb) and cadmium (Cd), i.e., typical harmful metals often found in water. Our model systems consist of a singly and doubly adsorbed neutral (${\mathrm{Pb}}^{0}$, ${\mathrm{Cd}}^{0}$) and charged (${\mathrm{Pb}}^{2+}$, ${\mathrm{Cd}}^{2+}$) atoms adsorbed on the GO nanoparticle of the chemical formula $C_{30}$$H_{14}$$O_{15}$. We show that a single charged metal ion binds more strongly than a neutral atom of the same type. Moreover, to determine the possibility of multiple adsorptions of the GO nanoparticle, two metal atoms of the same species were co-adsorbed on its surface. We found a site-dependent adsorption energy such that when two atoms of the same specie are adsorbed at sites $S_{i}$ and $S_{j}$, the binding energy per atom depends on whether one of the two atoms is adsorbed firstly on the $S_{i}$ or $S_{j}$ sites. Furthermore, the binding energy per atom for the two co-adsorbed atoms of the same specie (i.e., neutral or charged) is less than the binding energy of a singly adsorbed atom. This suggests that atoms may become less likely to be adsorbed on the GO nanoparticle when their concentration increases. We adduce the origin of this observation to be interplay between the metal–metal interaction on the one hand and GO–metal on the other, with the former resulting in less binding for the charged adsorbed metals in particular, due to repulsive interaction between two positively charged ions. The frontier molecular orbitals analysis and the calculated global reactivity descriptors of the respective GO–metal complexes revealed that all the GO–metal complexes have a smaller HOMO–LUMO gap (HLG) relative to that of pristine metal-free GO nanoparticle. This may indicate that although the GO–metal complexes are stable, they are less stable compared to metal-free GO nanoparticles. The negative values of the chemical potentials obtained for all the GO–metal complexes further confirm their stability. Our work differs from previous experimental studies in that those lacked details of the interaction mechanisms between GO, Pb and Cd, as well as previous theoretical studies which used limited numbers of parameters to characterise the GO–metal interactions. Rather, we present a set of parameters or descriptors which provide comprehensive physical and electronic characterisation of GO–metal systems as obtained via the DFT calculations. These parameters, along with those reported in previous studies, may find applications in rational design and high-throughput screening of graphene-based materials for water purification, as an example.

## 1. Introduction

With the industrial revolution, the issues related to the management and control of mining waste have increased. These mining wastes, mainly heavy metals, constitute a real source of pollution to the ecosystem since they are not generally biodegradable [1]. Present in soils, waters and air, heavy metals can also be found in many uses such as paints, dyes, in some fertilisers, soaps as well as in certain medicines [2]. Moreover, the bioaccumulation of metals such as Pb and Cb in the body is one of the causes of carcinogenic, cardiovascular and kidney diseases and can lead to death when absorbed in high concentrations [2,3].

Recent studies have indicated that two-dimensional nanomaterials containing oxygen, epoxy, hydroxyl and carboxylic groups [4] have a great potential for the adsorption of heavy metals [5,6,7,8,9,10]. In this context, graphene oxide (GO) nanoparticles have shown a great potential due to their high adsorption capacity, large surface area and solubility. These can also serve as an electrode material for monitoring contaminants [11]. They are hydrophilic in nature and have large negative charge surfaces that help to effectively remove cationic impurities such as heavy metal cations and cationic dyes by electrostatic interactions. Furthermore, due to their unique physico-chemical characteristics, they have the potential to become excellent adsorbents [12]. Indeed, Weijun et al. have shown that GO nanoparticles can be superior adsorbents for the removal of heavy metal ions from water [13]. They can also act as excellent catalysts or further hybridise with effective catalysts to convert harmful gases and organic species in wastewater [14,15]. Recently, many experimental works have been carried out in the field of wastewater treatment [9,16,17]. These include the use of treatment techniques to remove heavy metal ions from wastewater such as chemical precipitation, membrane separation, ion exchange, electrochemical treatment and metal adsorption. Out of all these techniques, metal adsorption has been found to show remarkable effectiveness. Moreover, due to its low cost, it is considered as a relatively cheap and fast approach for wastewater treatment. However, only few theoretical works have been reported on this topic [13,17,18,19].

At this point, it is important to detail some conclusions from previous experimental and theoretical studies, draw parallels between our work and these studies, and then emphasise how our work differs from them. In this regard, of note is the work of Sitko et al. [17], who performed experimental studies on the adsorption of divalent metal ions, including Pb and Cd, from aqueous solutions using graphene oxide as the adsorbent medium. While the work established the ability of GO to adsorb these metals from water solution, it did not provide the underlying atomic mechanisms or detail the electronic origin of the adsorption thus necessitating atomic studies. Similarly, Mashhadzadeh et al. performed a DFT study on the adsorption of atomic Ni, Cu, Cd and Ag on the surface of a zinc oxide nanotube and zinc-oxide graphene-like structure [20]. They concluded that while a ZnO nanotube can be a reasonable adsorbent for Cd, the ZnO–graphene sheet structure is unsuitable for a similar purpose due to low binding of the atom. However, their conclusion was based only on two physical descriptors, i.e., binding energy and equilibrium separation between the ZnO surface and the graphene. Thus, the study did not provide sufficient factors that may be used to comprehensively characterise the adsorption of the metals on the Zn-carbon based nanostructure. The work of Elgengehi et al. [19] examined the adsorption properties of Cd and Pb heavy metals on graphene and GO using the DFT theoretical investigation. However, the scope of their work is limited, mainly focusing on the calculations of density of states, adsorption energy and charge population analysis of metal–graphene and metal–GO systems.

In the present study, we have applied DFT calculations to investigate the structural and electronic properties of the GO nanoparticle having the chemical formula $C_{30}$$H_{14}$$O_{15}$ to Cd and Pb adsorption [4]. We consider the cationic state of the latter, i.e., ${\mathrm{Pb}}^{2+}$ and ${\mathrm{Cd}}^{2+}$, since they exist as such in an aqueous environment. However, for completeness, we also investigate the interactions of neutral atoms of these metals, i.e., ${\mathrm{Pb}}^{0}$ and ${\mathrm{Cd}}^{0}$ with the GO surface at the cationic adsorption sites. Moreover, we combine a set of descriptors to provide a comprehensive theoretical characterisation of the adsorbed metal–GO system. Specifically, we characterise the stability and reactivity of the metal on the GO by calculating the binding energy and performing frontier orbital analysis, molecular electrostatic potential (MEP) isosurface analysis and calculated Fukui functions. The latter, in particular, measures the chemical reactivity of a particular adsorption site to an external perturbation. Furthermore, we have used Mulliken charge analysis to probe the charge transfer between the GO substrate and the adsorbed metal atom. These analyses combined give insight into the atomistic mechanism underpinning the adsorption of the metal atoms, possible adsorption–desorption mechanisms, and provide scientific rationale for the observed experimental results [17]. Apart from building on existing studies, the present work, along with these previous works, may serve to provide useful and comprehensive descriptors for high-throughput screening of potential materials for water purification.

The outline of our paper is as follows: In Section 2, we present the computational details and atomic models used in this study. The fundamental structural and electronic properties of the GO nanoparticle are then presented in Section 3.1, which are followed by the adsorption and binding mechanism of Cd and Pb on GO as well as the underlying electronic interactions (Section 3.2).

## 2. Computational Details

**Figure 1:**
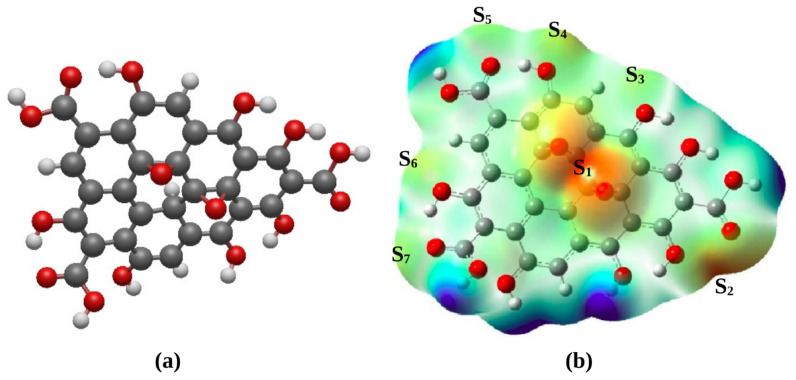
(**a**) Graphene oxide nanoparticle (GO). Carbon, hydrogen and oxygen atoms are represented as gray, white and red spheres. (**b**) Molecular electrostatic potential (MEP) isosurfaces of the GO nanoparticle. The blue colour indicates low electronic density, the white colour indicates medium electronic density, and the orange colour indicates high electron density region. S${}_{1}$, S${}_{2}$, S${}_{3}$, S${}_{4}$, S${}_{5}$, S${}_{6}$ and S${}_{7}$ are the most prominent sites for electrophilic attacks.

All the results presented in this work have been obtained with the density functional theory (DFT) as implemented in the Gaussian 16 package [21]. We used the Becke, 3-parameter together with the Lee–Yang–Parr (B3LYP) exchange-correlation functional, which is suitable for graphene-like systems, as well as the Los Alamos National Laboratory double zeta (LAN2dz) basis set for metal ions [22,23], and 6–31 g for the calculation of GO vibrational frequencies. Figure 1a shows the atomic model of GO nanoparticle used in the study. It has the chemical formula $C_{30}$$H_{14}$$O_{15}$ and is made up of 59 atoms which also contain carboxylic (−COOH), alcohol (−OH) and epoxy (−O−) chemical groups [4]. Moreover, the chemical composition of GO varies in a range from $C_{8}$$H_{2}$$O_{3}$ to $C_{8}$$H_{4}$$O_{5}$ while not considering groups coupled with graphene edges. This is consistent with the model adopted in this study. Moreover, based on the experimental data of Nakajima et al. [24], a model of GO structure which is intermediate between two ideal structures, $C_{8}$$O_{2}$ and $C_{8}$(OH)_4_, was proposed in line with $C_{30}$$H_{14}$$O_{15}$, i.e., $C_{8}$$H_{3.73}$$O_{4}$ which we have adopted in the present work. Most importantly, the GO nanoparticle has to be constructed in such a way that the overall charge is zero by passivating the residual dangling bonds with H atoms. To simulate the metal-adsorption behaviour of the GO nanoparticle, a metal atom M in a gas phase is placed on its most nucleophilic sites, which are denoted as $S_{1}$ and $S_{2}$. Each Pb or Cd (cation or neutral atom) is placed about 2.0 Å above (or beside) the GO surface. Such a separating distance is sufficient for the metal cation to interact with the GO nanoparticle. The full GO–metal system was optimised until all residual forces were less than $10^{-3}$ eV·Bohr^−1^. The placement of the metal cation at the nucleophilic $S_{1}$ and $S_{2}$ sites was informed by the fact that they have the highest electrophilic condensed Fukui functions [25,26,27,28], as will be shown in a later section of this paper.

It is worthwhile to state at this point that the condensed Fukui functions at a particular site measures its chemical reactivity to an external perturbation. Three different types of condensed Fukui functions for a particular atom or site *k* have been defined, depending upon the type of electron transfer,

(1) $$\begin{array}{c} f_{k}^{+}\left(r\right)=\rho_{{}_{N+1}}\left(r\right)-\rho_{{}_{N}}\left(r\right)\;\;\mathrm{for}\;\mathrm{nucleophilic}\;\mathrm{attack}, \end{array}$$

(2) $$\begin{array}{c} f_{k}^{-}\left(r\right)=\rho_{{}_{N}}\left(r\right)-\rho_{{}_{N-1}}\left(r\right)\;\;\mathrm{for}\;\mathrm{electroscopic}\;\mathrm{attack}, \end{array}$$

(3) $$\begin{array}{c} f_{k}^{0}\left(r\right)=\frac{1}{2}\left(\rho_{{}_{N+1}}\left(r\right)-\rho_{{}_{N-1}}\left(r\right)\right)\;\;\mathrm{for}\;\mathrm{radical}\;\mathrm{attack}, \end{array}$$

where $\rho_{N+1}\left(r\right),\;\;\rho_{N-1}\left(r\right)\;\;\mathrm{and}\;\;\rho_{N}\left(r\right)$ are the grosses electronic population of the atom or site *k*, calculated from the +1, −1 and 0 charged molecule. It should be noted that the greater the value of condensed Fukui function, the more reactive the particular atomic site in the molecule [27].

**Figure 2:**
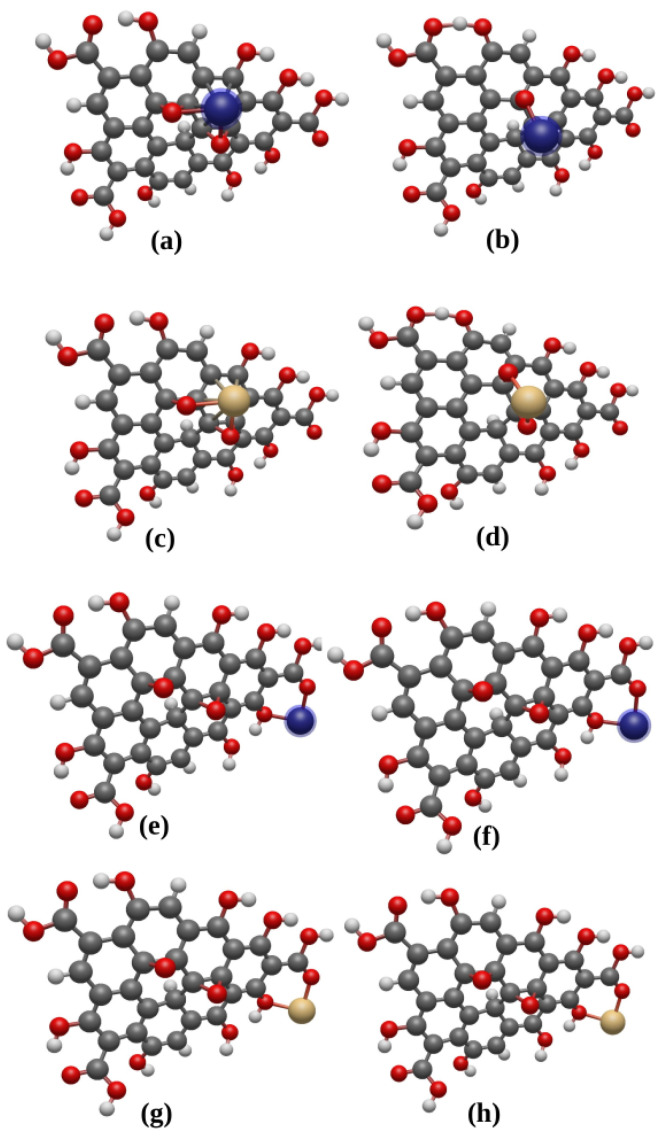
(**a**,**c**) GO nanoparticle with neutral atoms Pb${}^{0}$ and Cd${}^{0}$, respectively, adsorbed at site S${}_{1}$. (**b**,**d**) GO nanoparticle with cations Pb${}^{2+}$ and Cd${}^{2+}$, respectively, adsorbed at site S${}_{1}$. (**e**,**g**) GO nanoparticle with neutral atoms Pb${}^{0}$ and Cd${}^{0}$, respectively, adsorbed at site S${}_{2}$. (**f**,**h**) GO nanoparticle with cations Pb${}^{2+}$ and Cd${}^{2+}$, respectively, adsorbed at site S${}_{2}$. Carbon, hydrogen, oxygen, lead and cadmium atoms are represented as gray, white, red, blue and beige spheres.

Three separate models of metal-adsorbed GO have been considered. These are named as GO-M/$S_{1}$, GO-M/$S_{2}$ and GO-2M/$S_{1}$$S_{2}$. In the model termed as GO-M/$S_{1}$, a single metal M is adsorbed on the $S_{1}$ site (Figure 2a–d) while $S_{2}$ is the adsorption site of M in the model GO-M/$S_{2}$ (Figure 2e–h). In the case of GO-2M/$S_{1}$$S_{2}$, two metal cations of the same type occupy the adsorption sites $S_{1}$ and $S_{2}$. The structural model of GO-2M/$S_{1}$$S_{2}$ enables us to determine if the metal can be co-adsorbed at the two adsorption sites. To determine the minimum energy configuration for each of the three models, GO with metal cations ${\mathrm{Pb}}^{2+}$ and ${\mathrm{Cd}}^{2+}$ as well as GO with neutral metal atoms ${\mathrm{Pb}}^{0}$ and ${\mathrm{Cd}}^{0}$, each attached at their respective sites, were optimised. Note that neutral metal atoms, ${\mathrm{Pb}}^{0}$ and ${\mathrm{Cd}}^{0}$, have been investigated for the sake of comparison with the cations.

The chemical stability and reactivity were determined through the global reactivity descriptors using the frontier molecular orbitals, which are the HOMO (*Highest Occupied Molecular Orbital*) and LUMO (*Lowest Unoccupied Molecular Orbital*) with the Koopmans theorem [29]:

(4) $$E_{g}=E_{\mathrm{LUMO}}-E_{\mathrm{HOMO}},$$

(5) $$\mu =\frac{E_{\mathrm{LUMO}}+E_{\mathrm{HOMO}}}{2},$$

(6) $$\eta =\frac{E_{\mathrm{LUMO}}-E_{\mathrm{HOMO}}}{2},$$

(7) $$\omega =\frac{\mu^{2}}{2\eta },$$

where $E_{g}$, μ, η and ω are defined as the HOMO–LUMO gap (HLG), chemical potential, chemical hardness and global electrophilicity index, respectively. It has been demonstrated that a high HOMO energy corresponds to a more reactive molecule in the reactions with electrophiles, while a low LUMO energy favours molecular reactions with nucleophiles [30,31]. Note that a large HOMO–LUMO gap implies high kinetic stability and low chemical reactivity because it is energetically unfavourable to add electrons to a high-lying LUMO or to extract electrons from a low-lying HOMO [28,32,33,34,35,36]. A negative chemical potential suggests that a chemical compound or an atomic system is stable and will not decompose spontaneously into its constituent elements [37,38]. The chemical hardness may be interpreted as a measure of resistance toward the deformation of an electron cloud of a chemical system under small perturbations encountered during chemical process. The global electrophilicity index is related to the ability of an electrophile to acquire additional electronic charge and the resistance to exchange electronic charge with the environment [31,39,40].

Following the energy minimisation process, we calculated the binding energies per metal atom on the metal-free GO as well as the GO–metal complexes as:

(8) $$E_{b}=\frac{1}{n}\left(E_{n-1}+E_{a}-E_{n}\right),\;\;\;n=1,2,\ldots$$

where $E_{n}$ and $E_{n-1}$ are the minimum energies of GO nanoparticles having *n* and n−1 numbers of adsorbed metal atoms, respectively, while $E_{a}$ is the total energy of the isolated metal. In this convention, the positive value of $E_{b}$ means the adsorption process is favourable. Moreover, for a binding energy lower than 0.5 eV, the adsorbate (i.e., the metal atom) is generally considered to be physisorbed on the adsorbent. A higher binding energy indicates chemisorption [41]. Finally, to confirm that the optimised GO system is in the minimum energy configuration, which is chemically stable, we performed vibrational frequency calculations. It has to be noticed that imaginary frequencies in the vibrational modes will indicate an unstable molecule.

## 3. Results and Discussion

### 3.1. Stability and Reactivity of GO Nanoparticle

The molecule $C_{30}$$H_{14}$$O_{15}$ composed of 59 atoms has 174 normal modes of vibration. Depending on whether the vibration modes are given by the formula 3N- 6 (N is the number of atoms), the results of the frequency calculation have revealed no imaginary frequency, so we can deduce that our GO is stable (see Supplementary Information). Moreover, we have found that the paramagnetic ground-state is the preferred magnetic state for the GO nanoparticle, a result which is consistent with previous experimental and theoretical investigations of GO nanoparticles [42].

In Table 2, we present the calculated values for the HOMO–LUMO gap ($E_{g}$), chemical potential (μ), chemical hardness (η) and global electrophilicity index (ω). The HOMO–LUMO gap is found to be about 1.61 eV. It should be noted that chemical systems with a large $E_{g}$ are hard and much less polarisable compared to systems with a small $E_{g}$). A HOMO–LUMO gap (HLG) greater than 1.0 eV may be considered large [43]. Therefore, our model GO nanoparticle of relatively high $E_{g}$ can be considered to have a high chemical stability and chemical hardness, and thus will be less polarisable. Its electron cloud will be resistant to small perturbations, which will result in low chemical reactivity [31]. The chemical potential (η) is found to be −4.37 eV. This negative η also suggests that the GO nanoparticle is stable and does not undergo decomposition into elements. The chemical hardness of about 0.81 eV may be large enough to consider the GO nanoparticle relatively resistant toward the deformation of its electron clouds under small perturbations, which is further affirmed by the relatively high E${}_{g}$, i.e., greater than 1.0 eV. The global electrophilicity index of about 11.87 eV would be small enough to consider that the GO nanoparticle may be subject to electrophilic attacks.

In order to map out nucleophilic sites onto the GO nanoparticle, we plot the molecular electrostatic potential (MEP) isosurface. The plot is displayed in Figure 1b and shows variation in the electron density of GO nanoparticle, where the blue-coloured regions have low electronic densities while the white-coloured regions indicate medium electronic densities. The orange-coloured regions indicate high electron densities and may be considered as nucleophilic sites, and are thus potential sites for electrophilic attacks. Moreover, Figure 1b clearly shows that the most prominent nucleophilic sites are mainly localised around epoxy groups (site $S_{1}$) and the vicinity of carboxylic and alcohol groups (sites $S_{2}$, $S_{3}$, $S_{4}$, $S_{5}$, $S_{6}$, $S_{7}$).

For a better understanding of local reactivity properties, we calculated the condensed dual descriptors of atoms localised at the potential nucleophilic sites, defined as $f_{k}^{\left(2\right)}\left(r\right)=f_{k}^{+}\left(r\right)-f_{k}^{-}\left(r\right)$. The negative value of $f^{\left(2\right)}\left(r\right)$ indicates that the site is favoured for an electrophilic attack while the positive value indicates that the site may be favourable for a nucleophilic attack [25,26,27,28]. The calculated values of $f_{k}^{\left(2\right)}\left(r\right)$ are presented in Table 1, where we have demonstrated the variation of Fukui function values in the molecule by choosing the corresponding values for sites $S_{k}$ (*k* = 1–7). Here, $S_{k}$ (*k* = 1–6) have negative condensed dual descriptors while site $S_{7}$ has a positive condensed dual descriptor. The latter indicates that the electrophilicity of site $S_{7}$ prevails over its nucleophilicity, which may be explained by the proximity with a highly electrophilic site as shown by the MEP isosurface. Furthermore, the ranking of the electrophilic Fukui condensed functions of potential nucleophilic sites is as follows: $f_{1}^{-}\left(r\right)>f_{2}^{-}\left(r\right)>f_{4}^{-}\left(r\right)>f_{3}^{-}\left(r\right)>f_{6}^{-}\left(r\right)>f_{5}^{-}\left(r\right)>f_{7}^{-}\left(r\right)$. This clearly suggests that site $S_{1}$ has higher ability to attract electrophilic ions, followed by site $S_{2}$. For this reason, the following section will be devoted to the adsorption behaviour of both sites $S_{1}$ and $S_{2}$.

### 3.2. Adsorption of Pb and Cd

To determine the stability of metal adsorbates at the various sites, we calculated the binding energy $E_{b}^{i}$ {i=1,2} of adsorbed metal on sites $S_{1}$ and $S_{2}$ of GO nanoparticle using the relaxed minimum energy structures of GO-M complexes.

The resulting values are presented in Table 2. For a single metal adsorption, the $E_{b}^{i}$ is positive in all the cases, which indicates that the adsorption of a single Cd or Pb as a neutral atom or in ionic form is stable. Also to be noted from the table is that the ${\mathrm{Pb}}^{0}$ adsorption at the $S_{1}$ and $S_{2}$ sites of GO nanoparticle leads to a chemisorption with $E_{b}^{1}=5.93$ eV and $E_{b}^{2}=2.54$ eV, respectively. These are much stronger adsorption compared to ${\mathrm{Cd}}^{0}$, which is also chemisorbed at the $S_{1}$ site with a much lower binding energy of $E_{b}^{1}=1.55$ eV, but physisorbed at the $S_{2}$ site with $E_{b}^{2}=0.07$ eV. Our results are in agreement with theoretical investigations conducted by Elgengehi et al. [19] in the case of graphene-like material used as adsorbents for Cd and Pb heavy metals. They also found the same trend, namely, a neutral Pb atom absorbs more strongly than Cd. Furthermore, we observed a migration of neutral atoms (${\mathrm{Pb}}^{0}$ and ${\mathrm{Cd}}^{0}$) when adsorbed on site $S_{1}$, towards the closest hollow site, and these form covalent bonds with the neighbouring carbon atoms (see Figure 2). This behaviour indicates that the identified adsorption site $S_{1}$ for the ionic adsorbates is not a preferential site for neutral atoms.

The aforementioned results can be related to the Mulliken charge transfer between the GO nanoparticle and the adsorbed metal [44]. Table 2 presents different values of $\delta Q_{i}$ (where *i* is the site index), defined as the Mulliken charge difference between the electronic charge on the metal before and after adosorption.

(9) $$\delta Q_{i}=Q_{\mathrm{before}}-Q_{\mathrm{after}},$$

According to this definition, a negative $\delta Q_{i}$ implies that the metal adsorbate loses charges to the GO nanoparticle while the positive value corresponds to a gain of electronic charges by the metal. We observed higher charge transfers from ${\mathrm{Pb}}^{0}$ to the GO nanoparticle ($\delta Q_{1}=-0.98$ $e^{-}$ and $\delta Q_{2}=-0.60$ $e^{-}$) compared to those occurring from Cd${}^{0}$ ($\delta Q_{1}=-0.76$ $e^{-}$ and $\delta Q_{2}=0.04$ $e^{-}$), for the S${}_{1}$ and S${}_{2}$ sites, respectively. Thus, neutral Pb${}^{0}$ at the S${}_{1}$ and S${}_{2}$ sites acts as an electron donor while Cd${}^{0}$ at the S${}_{2}$ acts as an electron acceptor. Interestingly, Cd${}^{0}$ with a positive δQ has the lowest binding energy of all the single neutral metal atoms considered. Furthermore, the difference in charge transfers between Pb${}^{0}$ and Cd${}^{0}$ can be understood through the difference between their ionisation energies (IE) [45,46,47]. Neutral Pb and Cd have an IE of 7.42 eV and 8.99 eV, respectively [46]. Thus, it is easier to extract electronic charges from Pb${}^{0}$ compared to Cd${}^{0}$ due to the former’s lower IE. Hence, Pb${}^{0}$ transfers electrons more easily than Cd${}^{0}$ to the GO nanoparticle. Indeed, a similar trend in IE and charge transfer were obtained via first-principle investigations of adsorption properties of toxic heavy metals (i.e., Cd, Hg, Pb) on graphene quantum dots and infinite graphene structure [48].

We also calculated the harmonic vibration frequencies of both GO and GO-M with M being adsorbed on the S${}_{1}$ and S${}_{2}$ sites. The results show that both GO and GO-M are stable, while the vibration frequencies range from 25.8716 cm${}^{-1}$ to 3694.89 cm${}^{-1}$ for the GO nanoparticle and from 19.6641 cm${}^{-1}$ to 3722.71 cm${}^{-1}$, 24.8106 cm${}^{-1}$ to 3719.83 cm${}^{-1}$, respectively, for GO-Pb${}^{0}$ and GO-Cd${}^{0}$ concerning the adsorption on site S${}_{1}$, and from 15.9952 cm${}^{-1}$ to 3709.68 cm${}^{-1}$ and from 15.0921 cm${}^{-1}$ to 3711.59 cm${}^{-1}$ for GO-Pb${}^{0}$ and GO-Cd${}^{0}$ concerning site S${}_{2}$ (see Supplementary Materials for more details).

The adsorption of a single cationic adsorbate Pb${}^{2+}$ or Cd${}^{2+}$ at the S${}_{1}$ and S${}_{2}$ sites leads to a stronger chemisorption compared to those of a single neutral atoms (see Table 2). The binding energies of a single Pb${}^{2+}$ on the S${}_{1}$ and S${}_{2}$ sites are $E_{b}^{1}=12.33$ eV and $E_{b}^{2}=8.97$ eV, respectively. Moreover, Cd${}^{2+}$ is adsorbed with $E_{b}^{1}=12.18$ eV and $E_{b}^{2}=10.66$ eV at the S${}_{1}$ and S${}_{2}$ sites, respectively. These binding energies are an order of magnitude higher than those obtained for corresponding neutral Pb${}^{0}$ and Cd${}^{0}$ adsorptions as shown in Table 2. In general, charged metal ions, irrespective of the adsorption sites, bind more strongly compared to neutral atom of the same type. This result is in agreement with previous findings by Shtepliuk et al. [48]. Unlike the case of a neutral adsorbate where the atoms migrate to the preferential site on the GO surface, here, the metal cations Pb${}^{2+}$ and Cd${}^{2+}$ bound strongly with the oxygen atoms of epoxy groups at the nucleophylic site S${}_{1}$.

The Mulliken charge analysis shows a positive charge transfer, i.e., positive δQ, and thus electron transfer from the GO to the cations. Thus, each of Pb${}^{2+}$ and Cd${}^{2+}$ cations act as an electron acceptor from the GO nanoparticle. Therefore, positively charged ions reverse the direction of charge transfer compared to the charge-donating neutral atoms, which is consistent with previously reported results by Shtepliuk et al. [48]. We also investigated the adsorption of two neutral or charged Pb or Cd atoms on the S${}_{1}$ and S${}_{2}$ sites. Only co-adsorption of atoms of the same species are considered. Thus, we examined two models of adsorption, which are the following: (i) The first metal atom pre-exists at the the S${}_{1}$ site while the second atom adsorption occurs on site S${}_{2}$. Here, the binding energy of doubly adsorbed Pb or Cd is termed $E_{b}^{\;i,j}$ {(i,j)=(1,2)}, where (i,j)=(1,2) indicates that the metal atom pre-exists at site S${}_{1}$ before the adsorption of another atom of the same specie occurs at S${}_{2}$. (ii) The second adsorption occurs on the S${}_{1}$ site while the first one pre-exists on the S${}_{2}$ site. In this case, the binding energy of doubly adsorbed Pb or Cd is termed $E_{b}^{\;i,j}$ {(i,j)=(2,1), where (i,j)=(2,1) indicate that the metal atom pre-exists at site S${}_{2}$ before the adsorption of another atom occurs at S${}_{1}$. These binding energies per atom ($E_{b}^{\;i,j}$) are presented in the last column of Table 2. If these are compared to the binding energies of a singly adsorbed metal atoms ($E_{b}^{\;i}$), it is clear that the atoms become less binded to the GO nanoparticle with increasing number of adsorbed atoms. As an example, for a single Pb${}^{0}$ adsorption, $E_{b}^{\;i}=5.93$ eV, where for a doubly adsorbed atom of the same specie, $E_{b}^{\;i,j}=2.63$ eV. The same trend can be observed for all the metals, neutral or charged. In the case of Pb${}^{2+}$ ion, the binding energy per atom becomes negative (i.e., an unstable configuration or desorption) when the two charged ions are are co-adsorbed. It appears, therefore, that the adsorption characteristics of GO to metal ions is concentration- or coverage-dependent. It should be emphasised that adsorption behaviour may also depend on size and shape of the GO substrate as well as external factors such as solvation. However, these will not be discussed further as they are not the main focus of this work. It is, however, worthwhile to acknowledge these. Another observation from Table 2 is that the magnitude of $E_{b}^{\;1,2}$ is not the same as $E_{b}^{\;2,1}$. In all cases (for neutral and cations atoms adsorption), $E_{b}^{\;2,1}$ is larger than $E_{b}^{\;1,2}$. For example, $E_{b}^{\;2,1}=2.63$ eV for Pb${}^{0}$ while $E_{b}^{\;1,2}=0.94$ eV for the same atom specie. A similar trend is observed for all the atoms, as shown in Table 2. Thus, when a metal atom is initially adsorbed at the S${}_{2}$ site, it attracts and binds more strongly an atom of the same specie at the S${}_{1}$ site. In this case, the co-adsorption is thus energetically more favoured. However, when a metal atom is initially adsorbed at the S${}_{1}$ site, the second atom adsorbed on the S${}_{2}$ site experiences less binding. In fact, the co-adsorption may not be energetically favoured (that is, an unstable co-adsorption or desorption), as shown in the case of Pb${}^{2+}$ where $E_{b}^{\;2,1}$ is positive, i.e., 1.31 eV, whereas $E_{b}^{\;1,2}$ is negative, i.e., −0.37 eV. Thus, the lattice site of a pre-exisitng or an adsorbed metal atom on GO has a strong influence on subsequent binding or attraction of another atom of the same specie.

In order to gain deeper insight into the electronic origin of stability of adsorbed metal atoms on the GO nanoparticle, we performed frontier molecular orbitals analysis and calculated the global reactivity descriptors of the respective complexes (see Table 2). We found that the HLG of all GO-M complexes are smaller than that of pristine metal-free GO nanoparticle, which suggests that when GO adsorbs metal, the GO-M complexes are still energetically stable, although less stable compared to metal-free GO. Moreover, the lower HLG of GO-M complexes indicates that they are chemically soft, more polarized and have higher chemical reactivity compared to the GO nanoparticle. However, we found the chemical potentials to be negative for all the metal adsorption on both the S${}_{1}$ and S${}_{2}$ sites, suggesting that the resulting complexes are stable and may not spontaneously decompose into separate metal ions and GO nanoparticle. Further, the chemical hardness of GO–metal complexes are smaller relative to that of pure GO, which indicates that GO–metal complexes are less resistant toward the deformation of their electron clouds under small perturbations. Furthermore, metal adsorption on GO leads to higher global electrophilicity indexes compared to that of the metal-free GO nanoparticle. This indicates the reduction of the nucleophilic character of the GO nanoparticle following metal adsorptions.

Based on the analyses of the binding energy and frontier molecular orbitals, we conclude that, in general, multiple adsorption of metal atoms on GO is favoured, i.e., preponderance of positive binding energy for the co-adsorbed atom. However, as the number or concentration of adsorbed atom increases, the metal atom becomes less binded to the GO. Such a behaviour, in the case of charged ions in particular, may be rationalised as due to repulsive interaction between atoms of the same charge type, i.e., positive charge Pb${}^{0}$ and Cd${}^{0}$ ions. Indeed, Shtepliuk postulated that the competition between metal–metal interaction on the one hand and that of GO–metal on the other is the origin of less binding experienced by multiple metal atoms when adsorbed on the GO nanoparticle. Similarly, we may rationalise the attraction between metal ions when they are co-adsorbed to be due to strong perturbation of the electron cloud created by the adsorbed atoms, which results in bonding being established between the atoms. This will be consistent with Shtepliuk’s postulation of interactions between metal ions. These important observations will be useful to evaluate the adsorption and ion-retention capability of the GO nanoparticle as a medium to sequester heavy metal cations, in particular, those investigated in this work.

## 4. Summary and Conclusions

Density functional theory (DFT) calculations were employed to investigate the reactivity and interaction of a graphene oxide (GO) nanoparticle with neutral metal atoms Pb${}^{0}$ and Cd${}^{0}$ as well as charged Pb${}^{2+}$ and Cd${}^{2+}$ ions. Binding energy and global reactivity descriptors such as HOMO–LUMO gap (HLG), chemical potential (μ), chemical hardness (η) and global electrophilicity index (ω) were used to characterise the interaction and reactivity of the metal atoms with the GO nanoparticle. We demonstrated that our model GO nanoparticle is stable and has high chemical reactivity. We identified seven potential nucleophilic sites on the GO nanoparticle; however, only two of these were selected, due to their greater occurrences, to study the adsorption of metal atoms. Using a singly and doubly adsorbed neutral and charged metal atoms on GO as our model of GO–metal complexes, we found that the adsorption characteristics of charged metal ions, i.e., Pb${}^{2+}$ and Cd${}^{2+}$ differ significantly from those of neutral atoms Pb${}^{0}$ and Cd${}^{0}$. Firstly, we found that charged metal ions bind more strongly than neutral atoms of the same specie for single adsorptions. Moreover, we investigated the adsorption and binding capability of GO nanoparticles by considering model systems consisting of two Pb or Cd in a neutral or charged atoms. Only co-adsorption of atoms of the same type have been considered. We found site-dependency of the adsorption energy. Specifically, the binding energy of one atom at a nucleophilic site S${}_{i}$ when one other atom of the same specie is adsorbed firstly at another site S${}_{j}$ differs significantly to the binding energy of the same atom when the adsorption order on the two sites is reversed. Furthermore, it was observed that the binding energy per atom for two co-adsorbed atoms of the same specie is less than the binding energy of a singly adsorbed atom. This may suggest that with increasing number or concentration of the adsorbed atoms, they become less likely to be adsorbed on the GO surface. There is thus coverage dependence of adsorption behaviour of the metal ions on the GO. This observation may be ascribed to the metal–metal interaction in one hand and GO–metal on the other. The former, in particular, may results in less binding for the charged metal system due to repulsive interaction between two positively charged ions. We should, however, state that aside from metal ions coverage, the shape and size of GO substrate and the solvent medium in which the metal–GO system will interact may have some effects on the adsorption behaviour of GO. Calculations which take into consideration the effects of an explicit aqueous environment on the adsorption behaviour of metal–GO are currently being pursued and will be reported elsewhere. Furthermore, we performed frontier molecular orbitals analysis and calculated the global reactivity descriptors of the respective GO–metal complexes in order to gain deeper insight into the electronic origin of their stability. Our analyses revealed that all the GO–metal complexes have smaller HLG relative to that of pristine metal-free GO nanoparticle, which indicates that although the GO–metal complexes are stable, they are less stable compared to metal-free GO nanoparticles. In addition, we found the chemical potentials to be negative for all the studied GO–metal complexes, which confirms their stability. Finally, we note that in this work, we have not considered the inclusion of dispersion forces in the form of van der Waals (vdW) on the energetics of metal–GO systems. While indeed vdW may be important, our work nevertheless provides a basis for the rational design of GO for harmful metal ions capturing, which may be important in water purification when these are present as water pollutants.

## Figures and Tables

**Table 1:** Nucleophilic $f_{k}^{+}\left(r\right)$ and electrophilic $f_{k}^{-}\left(r\right)$ condensed Fukui functions, and the condensed dual descriptor $f_{k}^{\left(2\right)}\left(r\right)$ for different adsorption sites onto the free GO nanoparticle.

Structure	Site	$f_{k}^{-}\left(r\right)$	$f_{k}^{+}\left(r\right)$	$f_{k}^{\left(2\right)}\left(r\right)$
	S${}_{1}$	0.112	0.005	−0.106
	S${}_{2}$	0.087	0.051	−0.036
	S${}_{3}$	0.043	0.007	−0.036
GO	S${}_{4}$	0.053	0.012	−0.041
	S${}_{5}$	0.034	0.020	−0.014
	S${}_{6}$	0.036	−0.002	−0.038
	S${}_{7}$	0.027	0.028	0.001

**Table 2:** LUMO energy ($E_{L}$), HOMO energy ($E_{H}$), HOMO–LUMO gap ($E_{g}$), chemical potential (μ), chemical hardness (η) and global electrophilicity index (ω) of the GO nanoparticle and GO-M complexes (M = Pb${}^{0}$, Cd${}^{0}$). Mulliken charge transfers $\delta Q_{i}$ for the metal atom adsorbed at site S${}_{i}$ {i=1,2}. $E_{b}^{\;i}$ is the adsorption energy per metal atom adsorbed on the GO nanoparticle. Similarly, $E_{b}^{\;i,j}$ {(i,j)=(1,2),(2,1)} represents the binding energy per adsorbed metal atom when two atoms are co-adsorbed in such a way that the first atom is adsorbed at the S${}_{i}$ prior to the adsorption of a second atom at S${}_{j}$ site. In other words, a metal atom pre-exists at S${}_{i}$ before the second atom is adsorbed at S${}_{j}$.

Site	Structure	$E_{L}$ (eV)	$E_{H}$ (eV)	$E_{g}$ (eV)	μ (eV)	η (eV)	ω (eV)	$\delta Q_{i}$	$E_{b}^{\;i}$ (eV)	$E_{b}^{\;\mathrm{ij}}$ (eV)
	GO	−3.566	−5.176	1.61	−4.37	0.81	11.87	−	−	−
S${}_{1}$	GO-Pb${}^{0}$	−3.468	−3.764	0.30	−3.62	0.15	44.17	−0.98	5.93	2.63
	GO-Cd${}^{0}$	−3.851	−4.740	0.89	−4.30	0.44	21.25	−0.76	1.55	0.78
	GO-Pb${}^{2+}$	−9.879	−10.957	1.08	−10.42	0.54	100.68	0.86	12.33	1.31
	GO-Cd${}^{2+}$	−9.847	−10.808	0.96	−10.33	0.48	112.33	0.90	12.18	0.94
S${}_{2}$	GO-Pb${}^{0}$	−3.020	−4.246	1.23	−3.63	0.61	10.77	−0.60	2.54	0.94
	GO-Cd${}^{0}$	−3.673	−5.006	1.35	−4.34	0.68	13.88	0.04	0.07	0.04
	GO-Pb${}^{2+}$	−9.550	−10.366	0.82	−9.96	0.41	121.52	0.71	8.97	−0.37
	GO-Cd${}^{2+}$	−9.952	−10.563	0.61	−10.26	0.30	172.20	1.36	10.66	0.18

## Data Availability

The original contributions presented in the study are included in the article and Supplementary Material, further inquiries can be directed to the corresponding authors.

## Supplementary Materials

The following supporting information can be downloaded at: https://www.mdpi.com/article/10.3390/ma17122831/s1, Table S1: Modes and harmonic vibration frequencies of GO and GO-Pb${}^{0}$, GO-Cd${}^{0}$ for the sites S${}_{1}$ and S${}_{2}$.
