# Supplementary material for: Adsorption Behaviour of Pb and Cd on Graphene Oxide Nanoparticle from First-Principle Investigations
Source: Materials (Basel). 2024 Jun 10;17(12):2831. doi: 10.3390/ma17122831 (PMC11204970; doi:10.3390/ma17122831)
Supplement: Supplementary file 1 [file materials-17-02831-s001.zip › materials-3018869-supplementary.pdf]

materials

MDPI

Article

Adsorption Behaviour of Pb and Cd on Graphene Oxide Nanoparticle from First-Principle Investigations

Preslie Sala Nianga-Obambi [1], Dick Hartmann Douma [1,*], Anne Justine Etindele [2], Abdulrafiu Tunde Raji [3], Brice Rodrigue Malonda-Boungou [1,4], Bernard M'Passi-Mabiala [4] and Stephane Kenmoe [5,*]

1 Groupe de Simulations Numériques en Magnétisme et Catalyse, Faculté des Sciences et Techniques, Université Marien Ngouabi, Brazzaville BP 69, Congo; cohenobambi@gmail.com (P.S.N.-O.); malondabrice@gmail.com (B.R.M.-B.)
2 Higher Teachers Training College, University of Yaounde I, Yaounde P.O. Box 47, Cameroon; anne.etindele@univ-yaounde1.cm
3 Center for Augmented Intelligence and Data Science (CAIDS), College of Science, Engineering and Technology (CSET), University of South Africa (UNISA), UNISA Muckleneuk Campus, Preller Street, Muckleneuk, Pretoria 0003, South Africa; rajiat1@unisa.ac.za
4 Institut National de Recherches en Sciences Exactes et Naturelles (IRSEN), Brazzaville BP 2400, Congo; bmpassimabiala@gmail.com
5 Department of Theoretical Chemistry, University of Duisburg-Essen, Universität Str. 2, 45141 Essen, Germany
* Correspondence: dick.douma@umng.cg (D.H.D.); stephane.kenmoe@uni-due.de (S.K.)

check for updates

Citation: Nianga-Obambi, P.S.; Douma, D.H.; Etindele, A.J.; Raji, A.T.; Malonda-Boungou, B.R.; M'Passi-Mabiala, B.; Kenmoe, S. Adsorption Behaviour of Pb and Cd on Graphene Oxide Nanoparticle from First-Principle Investigations. *Materials* **2024**, *17*, 2831. https://doi.org/10.3390/ma17122831

Academic Editor: Abderrahim Yassar

Received: 3 May 2024
Revised: 30 May 2024
Accepted: 6 June 2024
Published: 10 June 2024

Copyright: © 2024 by the authors. Licensee MDPI, Basel, Switzerland. This article is an open access article distributed under the terms and conditions of the Creative Commons Attribution (CC BY) license (https://creativecommons.org/licenses/by/4.0/).

Supplementary Information

Table S1: Modes and harmonic vibration frequencies of GO and GO-Pb^0, GO-Cd^0 for the sites S_1 and S_2.

	Vibration Frequencies (cm^{-1})				
Modes	GO	GO-Pb^0/S_1	GO-Cd^0/S_1	GO-Pb^0/S_2	GO-Cd^0/S_2
1	25.8716	19.6641	24.8106	15.9952	15.0921
2	31.5981	27.4095	29.2052	28.9341	28.6341
3	43.3735	31.1224	30.7975	35.5836	33.8381
4	50.0603	38.8374	40.8256	48.0283	46.3759
5	55.846	47.5614	47.2508	54.9997	53.2376
6	65.8089	49.0654	49.9386	60.6926	55.1654
7	68.3883	61.4014	60.6612	67.4416	64.0575
8	95.5183	68.8719	69.8515	71.5416	69.7004
9	106.881	76.9066	77.9564	99.5659	80.115
10	108.394	90.9591	94.278	103.022	99.4707
11	116.631	100.861	101.401	106.437	105.338
12	139.072	101.044	106.871	110.689	106.469
13	151.311	106.478	107.838	126.521	113.535
14	161.334	140.142	132.554	139.658	126.133
15	172.218	149.523	144.005	149.506	146.897
16	184.462	152.211	151.149	157.118	154.74
17	193.469	169.046	172.6	170.319	168.527
18	209.217	177.647	175.202	184.496	182.413
19	218.615	191.917	182.574	186.787	185.413
20	224.173	199.638	190.95	201.743	196.914

21	234.943	211.49	207.473	221.136	218.905
22	250.86	221.536	214.533	229.928	228.368
23	256.936	228.742	222.03	238.9	238.798
24	262.537	236.307	229.975	254.413	254.693
25	265.06	244.822	240.358	259.429	256.951
26	280.581	254.655	251.17	262.097	258.76
27	295.949	266.464	263.28	270.433	271.076
28	309.104	276.798	268.162	280.775	281.539
29	311.989	286.78	279.239	294.607	285.036
30	322.811	301.914	304.296	300.553	298.854
31	327.357	312.644	308.906	310.745	308.947
32	342.7	322.066	316.917	323.052	322.943
33	352.145	324.527	325.814	327.655	324.753
34	355.844	333.835	329.175	330.912	329.029
35	365.615	339.223	340.428	342.782	339.879
36	367.521	349.102	340.963	353.692	352.531
37	372.46	353.188	357.321	354.858	356.125
38	384.164	358.468	361.233	359.465	359.389
39	397.273	369.238	364.947	368.459	367.115
40	406.046	370.725	370.165	373.375	372.448
41	410.652	388.884	385.864	384.603	381.626
42	419.587	394.923	393.156	391.955	391.734
43	421.998	400.617	398.365	404.51	402.778
44	445.611	414.869	409.308	410.089	409.476
45	449.57	422.476	419.2	419.204	421.168
46	459.955	436.383	434.532	427.439	437.677
47	468.694	438.349	438.771	436.656	442.785
48	487.928	455.225	451.772	446.192	448.939
49	497.731	463.176	456.791	450.755	462.189
50	503.947	468.481	471.805	460.716	466.779
51	513.084	475.697	480.357	466.365	472.892
52	529.18	496.937	496.607	484.682	487.017
53	540.126	506.665	498	498.188	494.071
54	544.199	526.381	519.225	505.12	503.117
55	555.427	533.882	524.739	512.507	509.716
56	574.641	539.34	538.053	529.425	527.793
57	583.974	547.251	543.66	533.182	531.65
58	589.466	557.47	552.381	545.238	544.354
59	591.828	562.384	560.225	552.516	551.304
60	601.345	574.508	575.295	563.445	567.361
61	609.069	586.151	585.789	573.975	574.812
62	615.865	589.985	588.55	576.224	579.933
63	620.668	596.846	597.203	584.155	590.405
64	629.546	603.816	598.54	591.682	595.165
65	638.519	612.145	608.071	593.365	596.406
66	654.647	623.55	624.734	598.024	605.986
67	669.074	632.813	625.853	618.837	617.098
68	674.338	639.529	635.613	628.395	626.546
69	682.61	643.929	649.348	646.578	646.639
70	686.878	661.042	658.768	652.139	651.559
71	701.779	668.13	668.854	653.903	655.813
72	706.823	673.757	676.166	671.299	670.316
73	714.467	682.414	680.902	672.016	671.948
74	719.829	691.089	683.959	685.436	687.885

75	732.586	701.7	691.907	691.163	692.708
76	748.192	709.669	700.75	704.6	705.14
77	757.346	723.472	720.471	709.964	710.029
78	765.573	730.056	728.525	721.543	719.221
79	773.993	739.219	740.027	734.516	735.1
80	776.93	747.131	750.365	736.571	737.705
81	788.108	752.068	765.479	744.319	753.041
82	795.834	758	766.465	761.368	759.153
83	797.988	767.323	768.6	772.321	771.167
84	808.325	769.621	783.31	775.164	775.548
85	811.496	779.698	790.412	784.83	782.565
86	817.322	799.474	801.695	787.373	788.773
87	826.061	801.52	821.695	791.448	790.992
88	834.836	817.366	827.344	796.577	795.25
89	854.695	826.529	831.98	815.973	818.46
90	857.257	835.578	836.892	824.471	824.237
91	865.249	842.876	841.587	830.229	828.459
92	869.043	846.319	845.189	843.989	843.081
93	874.133	848.731	850.868	851.51	848.851
94	901.77	850.39	861.927	866.297	863.799
95	913.729	870.315	867.231	870.232	867.328
96	922.169	876.127	869.749	886.57	882.71
97	952.54	878.461	877.681	909.207	908.636
98	961.451	890.598	891.064	937.285	921.521
99	975.063	910.494	910.292	941.749	923.942
100	996.57	942.744	942.35	948.422	941.549
101	1002.84	949.04	949.295	962.84	950.645
102	1024.78	963.922	967.411	970.085	956.48
103	1049.66	985.822	981.926	980.883	974.052
104	1066.92	999.545	997.649	987.771	980.296
105	1070.56	1018.56	1021.24	1019.36	1016.99
106	1096.36	1026.73	1027.37	1030.42	1028.31
107	1114.14	1060.01	1056.97	1044.41	1042.89
108	1130.98	1065.45	1063.24	1055.13	1050.02
109	1148.26	1070.74	1067.54	1084.49	1081.1
110	1165.33	1102.38	1100.59	1101.94	1098.43
111	1179.34	1106.91	1105.52	1111.92	1110.7
112	1192.52	1117.74	1113.09	1125.3	1126.43
113	1197.32	1128.33	1127.82	1147.07	1146.83
114	1213.35	1146.26	1137.88	1166.45	1166.33
115	1246.3	1155.81	1153.35	1177.96	1180.39
116	1257.79	1189.02	1183.44	1184.28	1183.74
117	1268.04	1196.58	1198.02	1189.82	1189.79
118	1279.74	1199.76	1199.26	1228.81	1220.26
119	1288.09	1228.32	1229.11	1239.76	1235.04
120	1301.77	1240.53	1244.3	1250.74	1250.7
121	1308.18	1258.07	1253.51	1261.52	1253.17
122	1312.36	1265.24	1260.15	1272.29	1269.8
123	1321.35	1272.09	1263.57	1286.91	1284.15
124	1327.06	1289.13	1291.37	1291.63	1290.22
125	1340.33	1294.02	1295.37	1310.79	1303.31
126	1343.19	1304.87	1302.52	1315.45	1313.32
127	1349.81	1316.33	1308.96	1319.3	1318.42
128	1361.33	1322.1	1327.41	1322.92	1320.59

129	1371.27	1333.76	1335.47	1337.04	1331.37
130	1379.74	1337.06	1340.03	1343.32	1340.11
131	1388.36	1340.36	1347.15	1345.21	1346.68
132	1393.58	1353.78	1353.12	1361.04	1360.26
133	1419.48	1358.18	1357.06	1365.23	1362.99
134	1426.65	1367.67	1368.48	1370.91	1377.1
135	1432.17	1380.58	1380.43	1394.13	1388.5
136	1435.82	1393.95	1392.89	1405.49	1391.6
137	1443.06	1398.66	1402.96	1408.36	1407.07
138	1463.04	1408.45	1406.82	1418.23	1411.91
139	1465.46	1421.91	1419.32	1423.92	1419.07
140	1480.37	1432.46	1426	1440.77	1435.34
141	1501.59	1441.1	1440.43	1445.02	1445.06
142	1508.94	1444.35	1445.37	1452.14	1447.12
143	1522.59	1460.01	1461.72	1456.6	1453.65
144	1544.05	1466.85	1477.62	1476.25	1473.37
145	1555.06	1492.11	1487.5	1493.99	1487.19
146	1568	1493.55	1491.54	1502.6	1497.35
147	1577.23	1504.62	1507.63	1532.17	1526.87
148	1584.51	1515.71	1512.53	1538.88	1532.42
149	1607.47	1580.18	1571.41	1554.58	1545.55
150	1613.01	1585.86	1575.93	1562.25	1553.84
151	1640.11	1600.65	1589.99	1572.82	1564.56
152	1658.79	1620.3	1606.18	1581.45	1575.87
153	1664.76	1629.06	1621.72	1593.78	1584.92
154	1667.8	1633.19	1631.54	1598.08	1595.53
155	1677.02	1648.11	1632.75	1623.58	1614.9
156	1684.63	1657.24	1648.91	1632.51	1626.34
157	1759.81	1672.64	1657.29	1641.55	1636.72
158	2890.71	1681.38	1667.01	1652.26	1649.5
159	3055.45	1698.05	1691.7	1668.8	1653.5
160	3095.44	1735.87	1736.37	1703.63	1692.46
161	3159.65	2938.33	2940.88	2883.08	2936.61
162	3259.63	3163.82	3164.23	2938.61	2996.66
163	3262.6	3228.93	3196.1	3059.15	3078.92
164	3280.61	3248.49	3204.19	3172.71	3169.28
165	3308.49	3253.72	3249.39	3257.72	3256.3
166	3387.63	3276.83	3253.94	3285.12	3285.84
167	3611.71	3291.06	3278.74	3305.12	3299.39
168	3626.9	3348.44	3305.1	3366.63	3333.2
169	3658.89	3404.97	3343.69	3401.12	3386.12
170	3672.4	3654.36	3653.82	3651.24	3651.32
171	3694.89	3660.33	3660.95	3659.46	3659.54
172	-	3685.2	3686.66	3703.82	3700.52
173	-	3701.5	3701.41	3708.58	3703.82
174	-	3722.71	3719.83	3709.68	3711.59
